# Pelagic Bacteria and Viruses in a High Arctic Region: Environmental Control in the Autumn Period

**DOI:** 10.3390/biology11060845

**Published:** 2022-05-31

**Authors:** Vladimir G. Dvoretsky, Marina P. Venger, Anastasya V. Vashchenko, Tatyana M. Maksimovskaya, Tatyana G. Ishkulova, Veronika V. Vodopianova

**Affiliations:** Murmansk Marine Biological Institute of the Russian Academy of Sciences (MMBI RAS), 183010 Murmansk, Russia; venger@mmbi.info (M.P.V.); an_nastasiay@mail.ru (A.V.V.); maximovskaya@mmbi.info (T.M.M.); ishkulova@mmbi.info (T.G.I.); vodopyanova@mmbi.info (V.V.V.)

**Keywords:** bacterioplankton, virioplankton, zooplankton, Barents Sea, Arctic Ocean, environmental influence

## Abstract

**Simple Summary:**

Microbial plankton represent a pivotal component of aquatic ecosystems worldwide. Despite their importance in pelagic ecosystems, microbial assemblages are less studied, in many Arctic regions. The study was concerned with the abundance of marine bacteria and viruses, in relation to a set of environmental variables, after the main productive season. We found a mosaic horizontal distribution of the microbial plankton, while the number of bacteria and viruses decreased with depth. Nutrients and zooplankton carbon were significant drivers of microbial abundance. Bacterioplankton abundance was positively correlated with counts of viruses, indicating close relations between these groups of microbes. Comparisons with previous studies revealed strong seasonal variations in the total abundance of marine bacteria, with the maximum values being in the summer and spring periods, while virioplankton counts were comparable in the autumn and spring–summer seasons. Our study is the first report regarding microbial plankton in the northeastern Barents Sea, in the autumn period, and provides baseline information, expanding current knowledge on the structure of pelagic Arctic ecosystems.

**Abstract:**

In the marine environment, bacteria and viruses play a significant role in carbon fluxes, remineralization processes, and the infection of various organisms. We performed a survey in the northeastern Barents Sea, a region adjacent to the Arctic Ocean, to investigate spatial patterns of microbial plankton, after the main productive period, in October 2020. Two main water masses occurred in the study region—colder Arctic Water and warmer Barents Sea Water, representing transformed Atlantic Water. Multivariate analyses detected patchiness in the horizontal distribution of bacteria and viruses, and their abundances showed no clear association with the water masses. There was an obvious vertical pattern in microbial concentration, with the highest estimates in the upper layers. Surface viral and bacterial abundance varied in a wide range (2.20 × 10^5^–10.7 × 10^5^ cells·mL^−1^ and 0.86 × 10^6^–14.98 × 10^6^ particles·mL^−1^, respectively) and were correlated with each other. Bacterioplankton was dominated by small-sized cells (<2 μm, 0.04–0.06 µm^3^), and the average volume of bacterial cells tended to increase toward the seafloor. The ratio of viral to bacterial abundance (VBR) was 11 ± 1 and did not differ between water masses and depth layers. VBR were higher, compared to summer values, suggesting a strong impact of viruses on bacterioplankton, after the main productive season. Redundancy and correlation analyses showed that inorganic nutrients (nitrate and phosphate) and organic carbon from zooplankton were most responsible for the total variability in the microbial parameters. Water temperature and salinity, also, had a measurable impact, but their influence was lower. Bacterial abundance was lower than in other seasons and regions of the Barents Sea, while viral abundance was comparable, suggesting a stronger viral impact on Arctic marine bacteria in the autumn season.

## 1. Introduction

Arctic ecosystems are very sensitive to changes in physical conditions and climatic variability; here, it is proposed to be more crucial, relative to lower latitudes [1,2]. There is a set of physical factors making the Arctic marine environment unique, namely low temperatures, ice cover, the presence of large shallow continental shelves, clear seasonal fluctuations in the light regime, and a high supply of freshwater discharge, originating from rivers and melting ice [2,3]. Strong climatic variations have been documented in the Arctic, since the end of the 20th century [3,4,5]. Climate forcing was reported to affect all trophic levels, from microbes to seabirds and marine mammals [5,6,7]. Studying ecological responses to climatic variation has a great significance in the sustainability of human activities and management of Arctic ecosystems.

The Barents Sea may be considered the most productive Arctic region [1,2]. The southern and southwestern areas represent the largest cod-fishery areas, located in the Arctic [1,2]. The Barents Sea is a transition zone, for Atlantic water entering the Arctic Ocean from the Norwegian Sea.

The pelagic ecosystem function and structure in the Barents Sea are, largely, determined by the hydrological regime, local circulation, frontal zones, seafloor topography, and general currents [1]. Recent climatic fluctuations had major impacts on marine ecosystems in the region, including northward expansion of boreal species, variability in the total productivity, and changes in the pelagic community structure [3,5]. Plankton assemblages play a key role in carbon fluxes, in ecosystems of the Barents Sea. Being sensitive to environmental influence, plankton communities are good indicators of climatic changes in Arctic seas [3,5,6,7].

In the pelagic ecosystems, bacterioplankton biomass is known to be a substantial fraction and, in some regions with low chlorophyll *a* concentrations, it can be higher than phytoplankton stocks [8,9]. Primary production and subsequent availability of dissolved organic matter promote heterotrophic bacterial growth and abundance [10]. Phytoplankton biomass is grazed upon by herbivorous zooplankton communities and, then, dead plankton as well as various excretes and metabolites are utilized by marine bacteria, serving microzooplankton (ciliates, tintinnids, and heterotrophic flagellates) as food resources [11]. Bacterioplankton assemblages are responsible for exchanging active metabolites (vitamin B12, antibiotics, and growth promoters) that may regulate algal–bacterial interactions [10,11]. Nutrient release to the seawater, due to decomposition of animals and plants, primarily by bacterial action as well as bacterial metabolites, is, thus, of great significance in allowing further photosynthetic cycles in the Barents Sea [1].

Viruses are abundant and active components of marine ecosystems, which, primarily, infect the numerically dominant bacterioplankton and eukaryotic marine microbes [12]. Being host-specific pathogens, they may affect bacterioplankton abundance and composition [13]. Viral lysis of host cells provides substrates for bacterioplankton and can modify energy and matter fluxes in the microbial plankton [12,13]. Therefore, the activity of marine viruses may be considered a significant factor, determining the productivity of higher trophic levels and the efficiency of organic carbon transfer, in the marine environment worldwide [14]. The abundance and distribution of marine microbes and their contributions to pelagic-food-web dynamics in polar waters have been, intensively, investigated during the past decades [15,16,17,18,19,20]. Information regarding the standing stocks of microbial plankton in the Barents Sea is, relatively, sparse. There are few studies dealing with bacterioplankton and virioplankton ecology in the region [21,22,23,24,25,26]. However, previous studies were focused on the western, central, and southern regions, in the spring and summer periods. The northern part of the Barents Sea is less studied and there are no data on the microbial plankton in the autumn period, after the main productive season.

The aims of the present study were to (1) describe the horizontal and vertical distribution of virioplankton and bacterioplankton, (2) test a hypothesis that microbial plankton is associated with different water masses, and (3) reveal which factors are responsible for spatial variations in microbial abundance and biomass, in the northeastern Barents Sea in autumn.

## 2. Materials and Methods

### 2.1. Sampling

**Figure 1:**
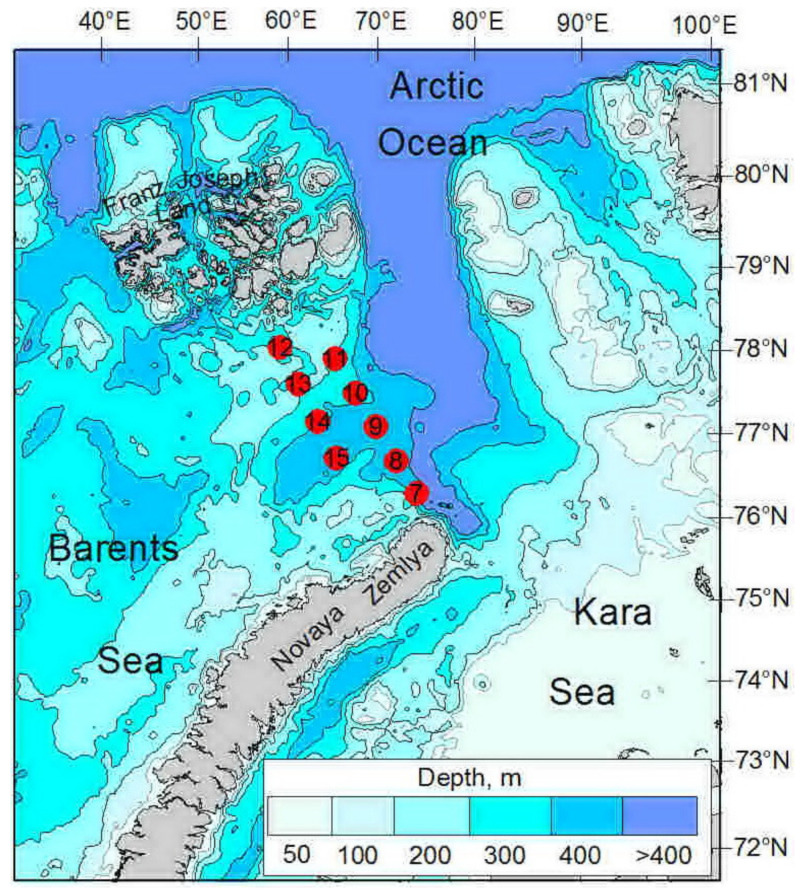
Location of sampling stations in the northeastern Barents Sea, in October 2020.

Water samples were collected at nine stations at two transects located in the northeastern Barents Sea, during a research cruise of the R/V *Dalnie Zelentsy*, between 8 and 11 November 2020 (Figure 1, Table 1). Vertical profiles of water temperature and salinity were recorded with a CTD Sealogger (SeaBird SeaCat SBE-19, CTD) [27]. Water masses were distinguished, based on hydrological criteria (Figure S1) [2,4]. Water samples were collected at 5–6 depths (0 m, 10 m, 25 m, 50 m, 100 m and near the bottom) at each station, using 10 L Niskin water bottles, attached to a rosette. Subsamples were taken from each bottle, for the determination of nitrate (NO_3_^−^), phosphate (PO_4_^3−^), and dissolved oxygen, as well as bacterial and viral abundance, for each sampling depth. Chlorophyll *a* concentrations were determined, for the upper 0 m and 10 m layers. Considering the significance of plankton animals, as a potential organic substrate for microbial growth and development, we collected zooplankton in the upper 100 m layer, using a Juday net (180 µm mesh size and 0.11 m^2^ mouth opening). Zooplankton sampling was performed, in accordance with our previous studies [28,29,30,31].

### 2.2. Processing

Samples for nutrient determination were collected into acid-cleaned polyethylene tubes, after thorough rinsing and filtration, through a 45 µm polycarbonate filter (FMPA, Vladisart, Vladimir, Russia) inserted in a filter holder. Samples for nutrient analysis were collected in 20 mL vials and, immediately, frozen in liquid nitrogen and, then, stored at −20 °C to further analysis. Determination of dissolved-oxygen concentration was carried out using the Winkler method with an automatic-endpoint-detection burette Digital Burette VITLAB (63762 Grossostheim, Bavaria, Germany). Analyses of dissolved inorganic nutrients were carried out at the Murmansk Marine Biological Institute laboratory, according to standard methods [32].

Water samples for chlorophyll *a* determination with a volume of 5 L for each sampling layer were filtered, immediately, through 0.6 µm Vladiopore filters. The filters were kept frozen until the onboard analysis. The filters were, subsequently, extracted in 90% acetone, placed in a freezer at 4 °C for 24 h, and centrifuged, following the standard procedure [33]. Chlorophyll *a* concentrations were measured, using a Nicolett Evolution 500 spectrophotometer (Spectronic Unicam, Scotia, NY, USA), which had been calibrated with commercially purified chlorophyll *a* preparations.

In the laboratory, the zooplankton organisms were identified and counted under an MBS-10 stereomicroscope. Abundance was expressed as individuals·m^−3^. Calculations of biomass of particular taxa were made using published mean individual wet, dry, or carbon weights as well as length/weight relationships [34,35,36]. All values were computed as mg carbon mass (DM) per cubic meter, using the following relationship: 1 mg wet mass = 0.2 mg dry weight = 0.1 mg C [37]. The conversion of wet to dry weight, for Ctenophora and medusae, assumed 1 mg wet mass = 0.04 mg dry weight [37].

Water samples for marine bacteria were preserved with particle-free 40% formaldehyde (final concentration of 2%) and, immediately, stored in slide boxes at −20 °C, for further microscopic analysis in the laboratory. Sub-samples (20 to 100 mL) were stained with DAPI for 8–10 min and filtered onto 0.2 µm Nuclepore filters using a low vacuum [38]. The blackened Nuclepore filters were supported by GF/F filters, during the filtration process, to facilitate a homogenous distribution of cells. Filtered samples were mounted on slides in immersion oil and stored at −30 °C, in preparation for microscopic examination. Microbial cells were enumerated, using an OlympusBX 53 epifluorescence microscope, with a barrier filter for UV-light excitation. Approximately 400 to 500 bacterial cells were counted per filter, using the 1000× objective. Microbial cells were fractionated into size classes: small single cells (<2 µm), ultra-small single cells (cell volume <0.04 µm^3^), large cells (>2 µm), and chain cells (cell length-width ratio ≥10). The dimensions of at least 30 small cells in each sample were measured, and the average value (ABV–average bacterial cell volume, µm^3^) was calculated. All cells >2 µm were counted, directly, and their biovolume was calculated, according to shape. The wet bacterial biomass was converted to carbon biomass (C, fg·cell^−1^), using the following equation: C = 120 × V^0.72^, where V–average cell volume, µm^3^ [39]. Virus-like particles were enumerated, after staining the samples with SYBR Green I fluorochrome (Molecular Probes, Eugene, OR, USA) [40]. Depending on the number of viruses, between 0.5–1.0 mL of seawater was filtered through 0.02-μm pore-size Anodisc aluminum oxide membrane filters (Whatman). Two filters were analyzed for each water sample, and a minimum of 400 viruses were counted, on each filter.

Contact rates (R) between viruses and bacteria at each sample were determined from the equation: R = (Sh × 2π × w × Dv) × V × P [41], where S is the Sherwood number (dimensionless), w is the diameter of a marine bacterium (0.45 × 10^−4^ cm; Ref. [42]), D is the diffusivity of viruses (3.456 × 10^3^ cm^2^·d^−1^), and V and P are the viral and bacterial abundances, respectively. Additionally, we calculated the ratio of viral to bacterial abundance (VBR).

### 2.3. Statistical Analyses

Differences in environmental parameters (temperature, salinity, nutrient and chlorophyll *a* concentrations, zooplankton abundance, and biomass) between different water masses and depth layers were tested using one-way ANOVA or the Kruskal–Wallis test and a post hoc Tukey test (*p* < 0.05). Assumptions of ANOVA were checked using a Kolmogorov–Smirnov test for normality and Levene’s test for homogeneity of variances. The Kruskal–Wallis test is the non-parametric analog of a one-way ANOVA, which does not make assumptions about normality. It is performed on the ranks of the measurement observations [43]. Non-parametric multivariate techniques were applied to microbial data using the PRIMER v5 software package [44]. Hierarchical cluster analysis was performed on the bacterioplankton and virioplankton abundance data. Prior to the analyses, the data were lg(x + 1)-transformed, to ensure the normality and homogeneity of variances. Similarities between the samples were assessed, using the Bray–Curtis index [45]. The relative contribution of each microbial cell size class and total virioplankton number to the dissimilarities between stations was assessed, by applying a one-way similarity percentage analysis (SIMPER; [44]). To determine the spatial variations, non-metric multidimensional scaling (nMDS) ordinations were computed and Analysis of Similarities (ANOSIM) was used, to detect significant (*p* = 0.001) differences between water masses and depth layers [45], with respect to microbial abundance. ANOSIM is sensitive to differences in multivariate dispersion. Therefore, to test the hypothesis of equality in group dispersions, we applied a multivariate analog to Leneve’s test. Homogeneity of dispersion among groups was calculated, as an average distance (±SE) of group members (samples) from the group’s centroid [46]. Comparisons of bacterial and viral abundances (ANOSIM), initially, showed no significant differences between the 0 m, 10 m, and 25 m layers and between the 50–100 m layers, although significant differences were found, between the 0–25 m and 50–100 m layers. Therefore, environmental and biological data were grouped into three groups, corresponding to the surface (0–25 m), intermediate (25–100 m), and bottom (192–365 m) layers.

To study the relationship between environmental variables (water temperature, salinity, sampling depth, nutrient concentrations, chlorophyll *a* content, zooplankton abundance, and biomass) and microbial data (bacterial abundance and biomass, viral abundance, ABV, R, and VBR), constrained ordination procedures were performed in the CANOCO for Windows v4.5 software [47]. The linear model (redundancy analysis, RDA) was chosen, after test runs of detrended correspondence analysis, which showed that the gradient length of the first axis was 0.235, suggesting that microbial data had linear microbial data–environment responses [47]. RDA represents a linear model, i.e., by linear combinations of environmental variables. Before including the selected environmental variables in the RDA, we tested our data for collinearity and, then, removed the variables that had a variance inflation factor higher than 10 (VIF > 10).

Ordination techniques represent multivariate methods that arrange sites along axes on the basis of biological data (species composition, abundance, biomass, etc.). The aim of this approach is to summarize multivariate data, in a convenient way, in scatter diagrams [47]. RDA is a canonical version of principal component analysis (PCA), of dependent variables Y (microbial parameters) constrained by independent variables X (environmental factors) [48,49]. The goal of RDA is to find the combination of independent variables that best explains the variation (or dispersion) of dependent variables. As in regression, each biological variable constitutes a response variable but, in ordination, these response variables are analyzed, simultaneously [47]. Ordination techniques are, widely, used in various studies to assess possible relationships between environmental factors (explanatory variables) and biological data [50]. In most studies, the RDA is used to reveal variations in biological communities, across a range of different environmental conditions. However, this method may be applied to the integrated parameters or several “traits” (e.g., total abundance, total biomass, and some others) [47]. In all cases, the primary data set contains records on a collection of observations–samples (sampling units) [47]. Each sample comprises measures for multiple species or, less often, the other kinds of descriptors (integrated community parameters) [47]. The primary data can be represented, as a rectangular matrix, where the rows refer to individual samples, and the columns indicate individual variables [47]. After log-transformation, the environmental and biological variables were, automatically, centered and standardized by the CANOCO program. Standardizing variables is the centering of the variables, around a mean of zero. Thus, the variables were converted to a z-score, so that the means were equal to 0 and the variance to 1 [49,50].

The forward procedure of non-collinear environmental variables in CANOCO was used, with a significance level of 0.05. The statistical significance of each environmental variable was evaluated, by Monte Carlo permutation tests [45]. RDA was supplemented, with the Pearson correlation analysis, to find possible significant correlations between biotic and environmental variables. The Benjamini–Hochberg procedure was used to correct *p*-values, for multiple testing. In each case, a false discovery rate was set at 0.1. Means are presented with standard errors (±SE).

## 3. Results

### 3.1. Environmental Parameters

**Figure 2:**
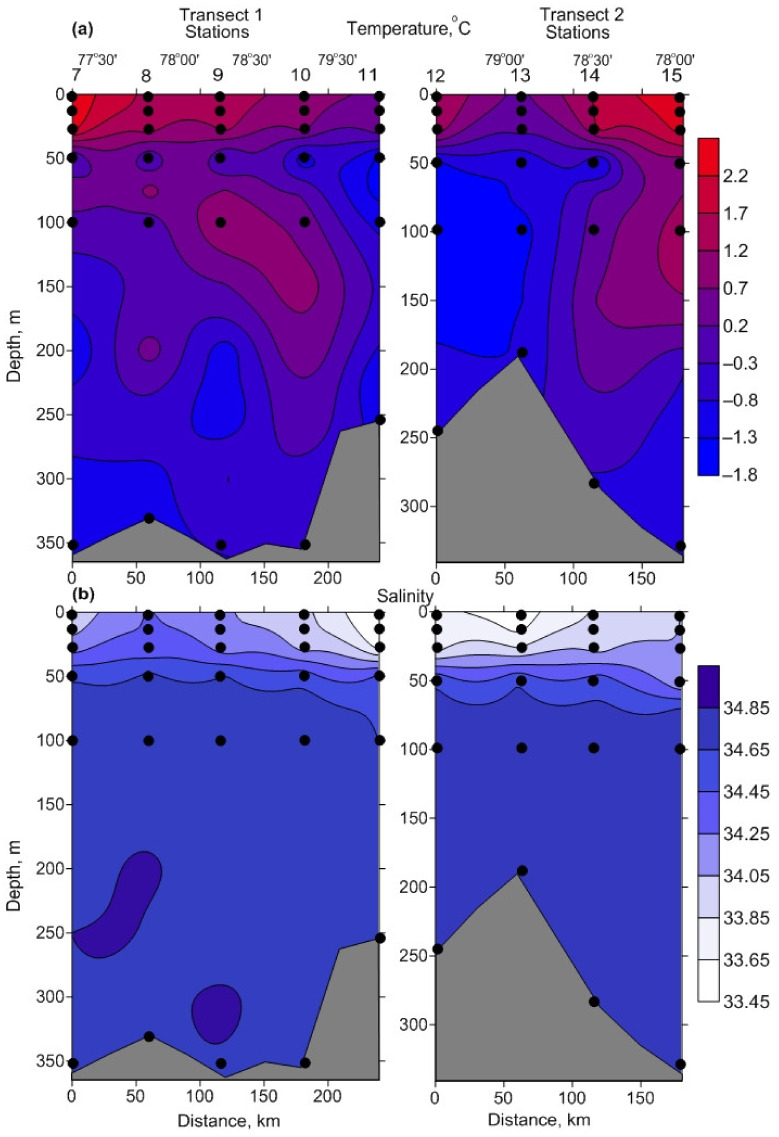
Vertical profiles of temperature, °C (**a**), and salinity (**b**) along the transects in the northeastern Barents Sea, in October 2020. Distance shows the intervals between sampling points.

Water temperature varied in a wide range (−1.65 °C to 2.51 °C) and the mean values tended to decrease, from the southeastern to the northwestern part of the study area (Table 1, Figures S1 and S2). The upper 10 m layer, at all stations, was characterized by positive temperatures, while below 50–100 m there were colder waters (<0 °C). The bottom layers were occupied by low temperature waters (−0.9 °C to −1.3 °C) (Figure 2). Only small changes in salinity were recorded over the study period. Salinity ranged from 33.49 to 34.84, with the lowest values in the surface (0–10 m) layers (Figure 2). There was an increase in salinity with depth. There was a weak pycnocline located at 30–60 m. Two main types of water masses were recorded in the study area (Figure S1). The Barents Sea Water (BSW), a water mass formed mainly from AW, as a result of the heat loss, was identified at two stations (St. 7 and 8) located near Novaya Zemlya. The Arctic Water (ArW) was present at the rest stations. There were significant differences in water temperature and salinity between the water masses and depth layers (one-way ANOVA or Kruskal–Wallis test, *p* < 0.05), except for the bottom layers (Table 2). The sea was ice-free during the study period.

Nutrient concentrations decreased from the southern to the northern part of the study area (Table 2). This trend was, particularly, evident for phosphate and nitrate. Phosphate concentrations ranged from <0.01 µM to 0.89 µM, while nitrate concentrations varied, from 0.1 µM to 18.65 µM. Vertical distributions of these nutrients showed an increase in the deepwater layers (Table 2). Mean oxygen concentrations demonstrated low horizontal variability (6.81–8.20 mL·L^−1^), although there was a clear vertical trend, with the lowest values being recorded below 50–100 m (Table 2). One-way ANOVA revealed significant differences (*p* < 0.001) in phosphate and oxygen concentrations, between water masses and depth layers, while nitrate concentrations were similar in both water masses but differed regarding sampling layers (*p* < 0.001). Phosphate and nitrate concentrations were significantly positively correlated with salinity and negatively correlated with water temperature (Pearson’s correlation, Benjamini–Hochberg *p* < 0.1). In contrast, oxygen concentrations tended to increase with temperature and decrease with salinity (Pearson’s correlation, Benjamini–Hochberg *p* < 0.1).

Surface chlorophyll *a* concentrations varied between 0.18 and 0.36 mg·m^−3^ and were, relatively, homogeneous in the study area (Table 2). No clear relationships (Pearson’s correlation, Benjamini–Hochberg *p* < 0.1) were detected between temperature, salinity, nutrients, and chlorophyll *a* concentrations.

**Figure 3:**
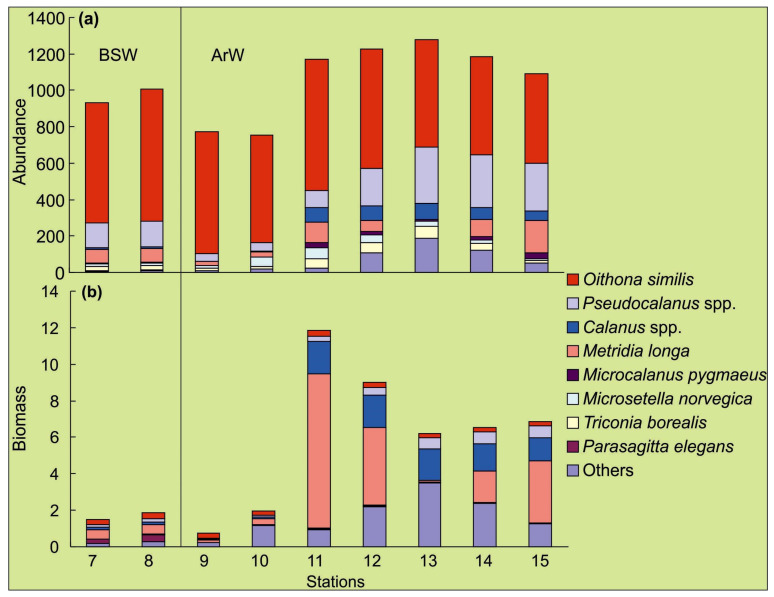
Zooplankton composition, abundance ((**a**)—individuals·m^−3^) and carbon biomass ((**b**)—mgC·m^−3^) in the 0–100 m layer in the northeastern Barents Sea, in October 2020. Water masses: BSW—Barents Sea Water, ArW—Arctic Water.

Zooplankton density ranged between 752 ind.·m^−3^ (st. 10) and 1278 ind.·m^−3^ (st. 13), averaging 1012 ± 87 ind.·m^−3^ (Table 2, Figure 3). The total zooplankton biomass varied from 0.8 to 11.8 mgC·m^−3^, with a mean value of 4.9 ± 1.7 mgC·m^−3^ (Table 2, Figure 3). Both parameters increased along Transects 1 and 2 northwestward. Significant spatial differences were found, regarding the total zooplankton biomass, with the highest estimates in ArW (one-way ANOVA, *p* < 0.001) (Table 2, Figure 3). Copepods were the most diverse and numerous group, accounting for 94–99% of the total abundance and 40–92% of the total biomass (Figure 3). The zooplankton assemblage of BSW was characterized by a high proportion of small copepods (*Microcalanus pygmaeus*, *Microsetella norvegica*, *Oithona similis*, *Triconia borealis*, *Pseudocalanus* spp.), in the total zooplankton biomass (Figure 3), while larger species (*Calanus* spp. and *Metridia longa*) amounted to >50% of the total biomass in ArW (Figure 3). The total zooplankton biomass was found to be positively correlated with surface chlorophyll *a* (Pearson’s correlation, Benjamini–Hochberg *p* < 0.1).

### 3.2. Spatial Variations of Microbial Plankton

**Figure 4:**
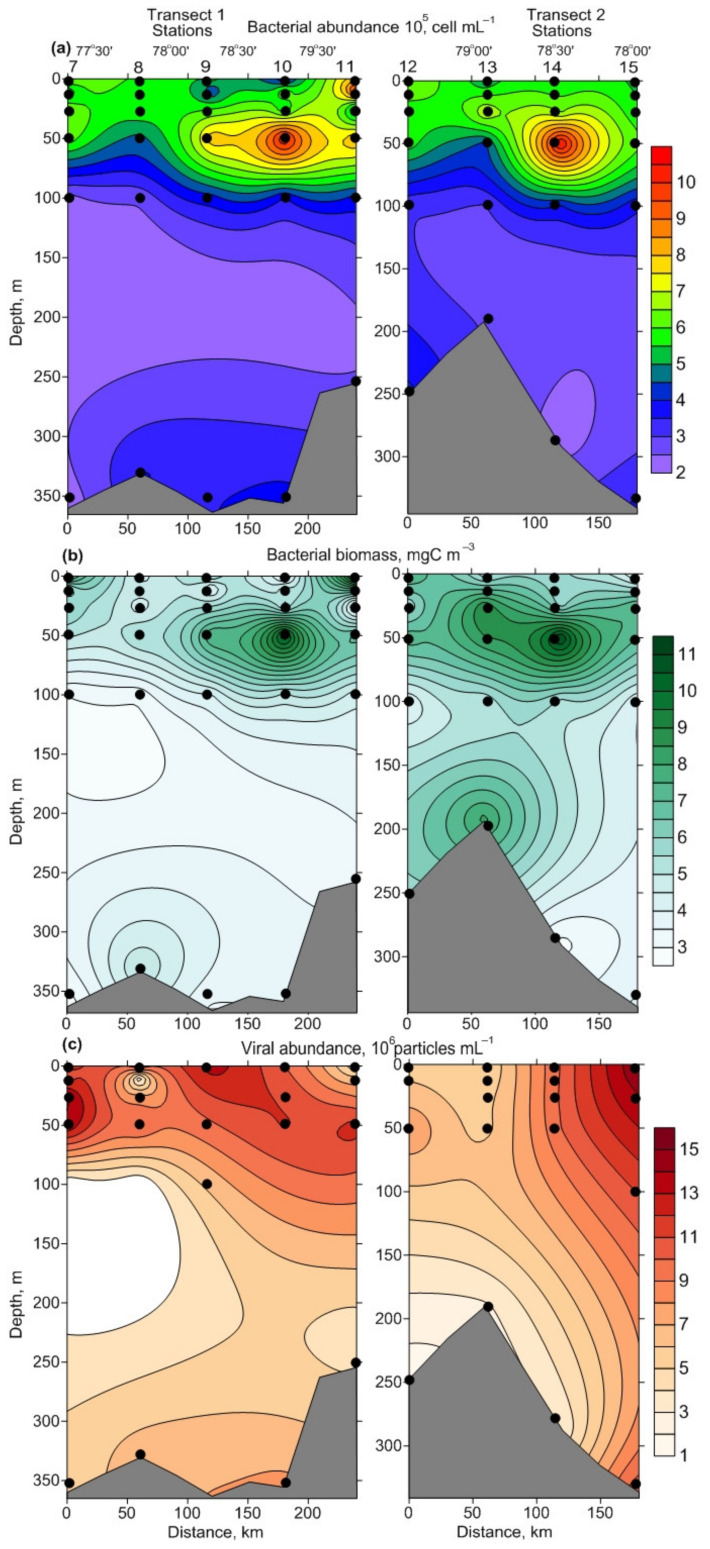
Vertical profiles of bacterial abundance ((**a**), 10^5^ cells·mL^−1^), bacterial biomass ((**b**), mgC·m^−3^), and viral abundance ((**c**), 10^6^ particles·mL^−1^), along the transects preformed in the northeastern Barents Sea, in October 2020.

**Figure 5:**
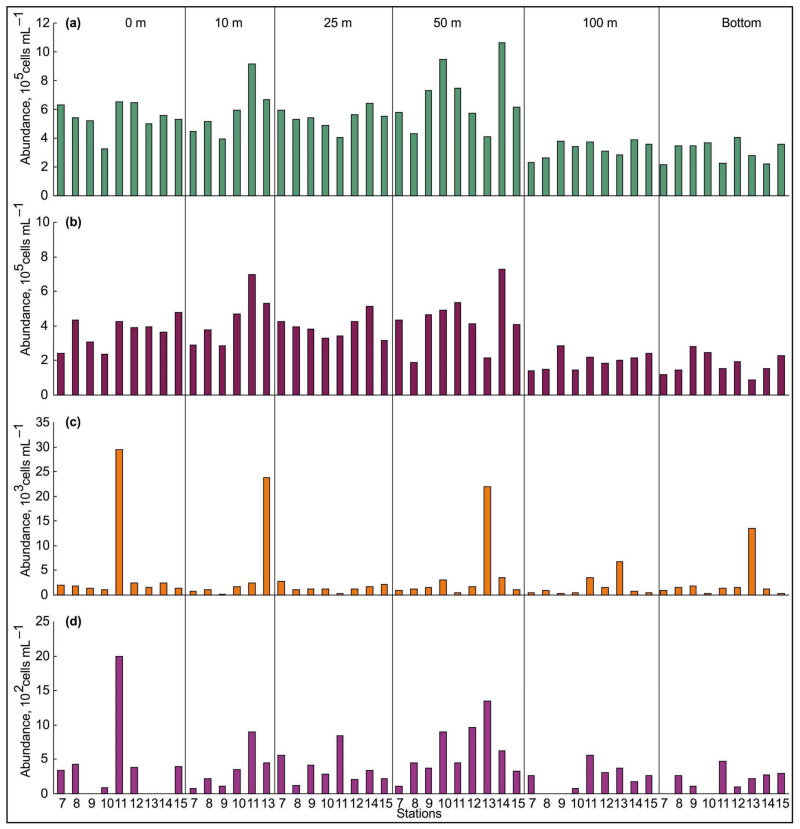
Abundance of bacteria ((**a**)—small, (**b**)—ultra small, (**c**)—large, (**d**)—chain cells) at each station in the northeastern Barents Sea, in October 2020.

The bacterial abundance varied from 2.20 × 10^5^ to 10.68 × 10^5^ cells·mL^−1^ (Figure 4a), with an average of 4.97 ± 0.27 × 10^5^ cells·mL^−1^. The surface and bottom counts increased from the Novaya Zemlya coast to Franz Joseph Land (Figure 4a). Mean values detected in BSW were comparable with those found in ArW (Table 2), although there were significant differences in the total bacterial abundance between the 0–100 m and bottom layers (Kruskal–Wallis Test, *p* < 0.001). The abundance showed two peaks at the intermediate layer (50 m) as well as stations 10 and 14 (Figure 4a). The bacterial biomass varied from 2.70 mgC·m^−3^ to 12.48 mgC·m^−3^ (mean 5.90 ± 0.33 mgC·m^−3^) and demonstrated mosaic distribution within the study area, although maximum records were found at stations affected by ArW (Figure 4b). Small and ultra-small bacterial cells were the most numerous in the total bacterioplankton, comprising 98.7–99.7% (Figure 5a,b). Large bacterial cells accounted for 0.1–5.1% in the bacterioplankton and tended to be concentrated in the surface layer (Figure 5c). Chain-bacterial cells were, rarely, found in the samples and their proportion, in the total bacterial density, did not exceed 0.3% (Figure 5d). They reached maximal abundance in the intermediate layer. ABV ranged from 0.020 μm^3^ to 0.136 μm^3^ and tended to be slightly higher in ArW, relative to BSW, although the differences were non-significant (one-way ANOVA, *p* > 0.05) (Table 2).

Viral abundance ranged from 0.86 × 10^6^ particles·mL^−1^ to 14.98 × 10^6^ particles·mL^−1^, averaging 6.10 ± 0.45 × 10^6^ particles·mL^−1^ (Figure 4c). There were no significant differences between BSW and ArW, in the total number of viruses (one-way ANOVA, *p* > 0.05), but the surface and intermediate layers had greater values (Kruskal–Wallis Test, *p* < 0.001). The viral abundance was positively correlated with the bacterial abundance and biomass (Pearson’s correlation, Benjamini–Hochberg *p* < 0.1). VBR was estimated to be 1–28 (11 ± 1) (Table 2). This variable did not differ between water masses and depth layers (one-way ANOVA or Kruskal–Wallis Test, *p* > 0.05). Contact rates (R) between viruses and bacteria were 0.3–9.6 (3.5 ± 0.3) (Table 2) and significantly decreased with depth (Kruskal–Wallis Test, *p* < 0.001). Pearson correlation analysis found significant positive correlations of R, with viral and bacterial abundances (Pearson’s correlation, Benjamini–Hochberg *p* < 0.1), while R was negatively correlated with ABV (Pearson’s correlation, Benjamini–Hochberg *p* < 0.1).

**Figure 6:**
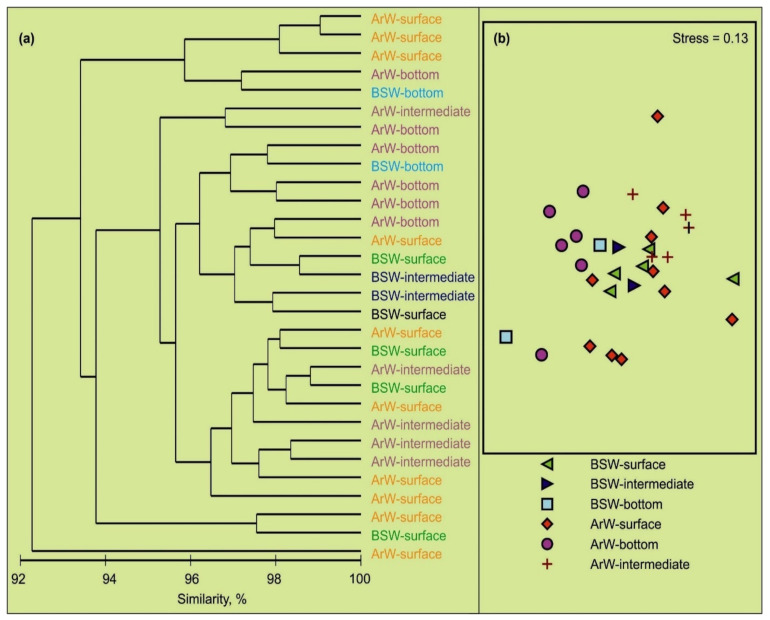
Cluster dendrogram (**a**) and NMDS ordination plot (**b**), showing relationships between stations, based on microbial parameters in the northeastern Barents Sea, in October 2020. Water masses: BSW—Barents Sea Water, ArW—Arctic Water.

Multivariate analyses (hierarchical cluster analysis and NMDS ordination), based on the abundance of microbial plankton, revealed a high degree of similarity between the two types of water masses in the region (Figure 6). The ANOSIM showed significant differences in the bacterioplankton and virioplankton abundances, between different sampling layers (global R: 0.195, *p* = 0.013), although there were no significant differences in the microbial density averaged for water masses (global R: −0.161, *p* = 0.974). Based on the SIMPER analysis, dissimilarities ranged from 19.9% to 44.4%. The highest dissimilarity occurred for the intermediate and bottom layers. The SIMPER analysis, also, indicated that viral abundance contributed, mostly (92–93%), to the total dissimilarities between sampling layers.

### 3.3. Environmental Control of Microbial Plankton

**Figure 7:**
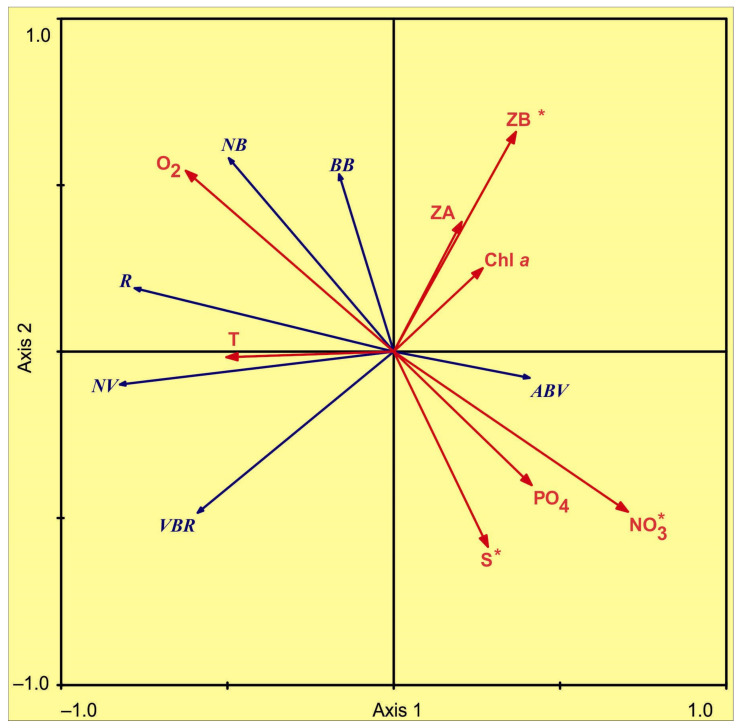
Redundancy analysis (RDA) on lg(x + 1)-transformed microbial abundance, indicating the ordination of microbial parameters and environmental variables in the northeastern Barents Sea, in October 2020. NB—bacterial abundance (10^5^ cells·mL^−1^), BB—bacterial biomass (mgC·m^−3^), NV—viral abundance (10^6^ particles·mL^−1^), VBR—the ratio of viral to bacterial abundance, ABV—average bacterial cell volume, R—contact rate between viruses and bacteria. T—temperature (°C), Sal—salinity, Chl *a*—surface chlorophyll *a* concentration (mg·m^−3^), PO_4_—phosphate concentration (µM), O_2_—dissolved oxygen concentration (mL·L^−1^), NO_3_—nitrate concentration (µM), ZA—zooplankton abundance in the upper 100 m layer (ind.·m^−3^), ZB—zooplankton biomass in the upper 100 m layer (mgC·m^−3^). Significant variables (forward selection procedure, Monte Carlo permutation test, *p* < 0.05) are marked with asterisks.

Possible relationships, between the microbial plankton and the environmental dataset, were analyzed using RDA analysis and supplemented with the Pearson correlation analysis (Figure 7, Table 3 and Table 4). The first two RDA axes (eigenvalues 0.480 and 0.127) explained 48.0% and 12.7% of the total variability in the microbial parameters, during the study period. The first RDA axis was significantly positively correlated with nitrate content and negatively correlated with water temperature and oxygen concentration (Figure 7).

The second axis was significantly positively correlated with zooplankton biomass and oxygen concentration and negatively correlated with salinity (Figure 7). The forward selection of environmental factors, using a Monte Carlo permutation test (999 permutations), showed that zooplankton biomass, nitrate concentration, and salinity were the factors that contribute significantly to the observed variability in microbial-plankton variations (Figures S2–S11 and Figure 7, Table 3). The three environmental variables together explained 45% of the total variance in microbial parameters (Table 3). The high bacterial abundances and biomasses were associated with low nitrate and phosphate contents as well as high oxygen concentrations (Figure 7, Table 3). The total viral abundances were connected with high temperature and low zooplankton biomass (Figure 7, Table 3 and Table 4). VBR tended to decrease with increasing zooplankton biomass (Figure 7, Table 3 and Table 4). ABV was positively correlated with nitrates (Figure 7, Table 3 and Table 4). High R was associated with low phosphate and nitrate as well as high oxygen concentrations (Figure 7, Table 3 and Table 4).

## 4. Discussion

The present study aimed to fill the gap in our knowledge of microbial plankton, in a less studied region strongly affected by cold waters from the Arctic Ocean, after the main productive season. Our data is the first report describing spatial patterns in bacterioplankton and virioplankton, in the northeastern Barents Sea. In general, we revealed that microbial abundance was strongly affected by a set of environmental variables (nutrient concentrations, zooplankton carbon biomass, and hydrological conditions).

### 4.1. Environmental Parameters

A clear warming trend has been detected in the Barents Sea, since the 2000s, and the mean water temperature was found to have risen [1,2,3,4]. In particular, in most regions of the Barents Sea, during the period of 1998–2017, the highest values of the mean annual surface temperature were registered in regions affected by warm AW, while the lowest estimates were in the northeastern Barents Sea [3]. Remote sensing data suggest that a 1.0 °C increase over the 20-year period was evident for the region [3]. We revealed that water temperature in the upper 0–50 m layer was somewhat higher, relative to the mean values recorded in October (1.0–1.2 °C vs. −1–0 °C), based on observations from 1950 to 1998 [51]. Moreover, in temperate and cold years, the region of Franz Joseph Land is covered by ice, but there was no sea ice during our cruise. This finding is in line with a general trend of ice loss, in the Arctic Ocean and adjacent areas [1,3,4]. Surface salinity, recorded in October 2020, was slightly higher, compared to the mean values obtained in 1950–1998 [51], suggesting a lower impact of cold ArW.

Nutrient concentrations demonstrate strong seasonal and spatial fluctuations in the Barents Sea [1,2,52]. Total depletion of phosphate and nitrate may occur in the upper layers during the spring–summer period, due to phytoplankton bloom in the Arctic [1,2,53,54]. Spring bloom leads to a decreased stock of nutrients, and in ArW of the Marginal Ice Zone, the outburst may occur earlier than in AW [54]. For instance, nitrate and phosphate concentrations in ArW (central Barents Sea), during spring bloom, were 2–3 μM and 0.2–0.3 μMm, respectively, while these in AW were 5–6 μM and 0.5–0.6 μM, respectively [55]. In the eastern Barents Sea, nitrate and phosphate concentrations in spring were 0.27–0.30 µM and 1.34–3.81 µM, respectively [53]. In the western Barents Sea, a clear seasonal cycle of nitrate has been documented, with a maximum of 9–10 µM in March and a minimum of ~1 µM in August [56]. In more northern regions of the Barents Sea, a peak of blooming is, typically, recorded in July–August [1]. In ArW, there is a rise in nutrient supply from late summer to winter, when nitrate and phosphate reach their maximum values [1].

In our study, we recorded a clear vertical pattern in the nutrient concentrations, with the richest waters being located in deeper layers. This pattern can be explained by intense vertical mixing processes, in the upper 50 m layer, and convection, as revealed in earlier studies [1,2]. Enhanced bottom concentrations of nutrients seemed to be associated with decreased water exchange, at deepwater sites (e.g., St. Anna Through), relative to intermediate and surface waters. Phytoplankton, also, play an important role in the regulation of overall nutrient stocks in the Arctic. The autumn stage of phytoplankton succession in the high Arctic regions may be specified with decreased abundance and biomass of pelagic microalgae [1,54]. Autumn–winter chlorophyll *a* concentrations are low and do not exceed 0.1–0.5 mg·m^−3^ [1,26,54,56]. We, also, revealed low surface chlorophyll *a* values, which confirmed this general pattern, for phytoplankton density to decrease, after the main productive season in the northern Barents Sea.

Our study found that zooplankton assemblages were dominated by copepods. The zooplankton composition was typical for ArW and BSW with *Calanus* spp., *Pseudocalanus* spp., *Microcalanus pygmaeus*, *Oithona similis*, and *Metridia longa* being common taxa [2,28,30,57]. Moreover, these species are among the most frequent and abundant zooplankters in other Arctic regions, including Svalbard waters, the northern Kara Sea, and Greenland waters [1,2,28,29,30,31,58]. We observed an increase in the total mesozooplankton biomass in ArW, compared to BSW, and this pattern was associated with the prevalence of small taxa in BSW. Larger copepods have a higher relative abundance in ArW, and the mean biomass here is 3.7 times higher than in BSW. Zooplankton abundance and biomass estimates in the present study were lower, when compared to the values recorded in ArW in the Kola Transect (33°30′ E), in the autumn–winter periods of 2011–2012 [30], and these differences can be explained by sampling methods and climatic conditions. Our study was focused on the upper 100 m layer, while the data obtained in 2011–2012 considered the whole water layer, plus the period of 2020 was colder, relative to 2011–2012 [3,46]. Local variability of hydrological conditions and water circulation might, also, be responsible for differences in the zooplankton density [1,2,29,30]. We, also, established a positive correlation between the zooplankton biomass and surface chlorophyll *a*. This result is expected because most species in our study were found to be herbivorous or omnivorous, and phytoplankton, apparently, played an important role in their feeding in the Barents Sea and other Arctic regions [1,2,59].

### 4.2. Spatial Variation of Microbial Plankton

In the high Arctic, prolonged periods of low water temperature, ice coverage, and darkness in the autumn–winter season lead to strong seasonal differences in bacterial abundance and biomass as well as concentrations of viruses [15,18,19,60]. In late spring, ice melting and increasing light irradiance promote phytoplankton bloom, which causes a subsequent rise in the abundance of Arctic microbial plankton.

Many studies have reported marked spatial and seasonal variations, in microbial characteristics in the Barents Sea. Spring and summer patterns of bacterioplankton have been investigated in the Marginal Ice Zone and in the northern Barents Sea [21,22,23,24,25]. The total abundance of bacteria was recorded to be 0.41 × 10^6^–4.1 × 10^6^ cells·mL^−1^, during summer in the central Barents Sea, and these counts were lower than the estimates reported for the northern Barents Sea, during the spring bloom [22] and summer periods [23]. Other studies found that the number of marine bacteria tended to be higher in AW compared to ArW in the late summer period [24,25]. There is only one study that reported autumn values of bacterioplankton abundance and biomass [26]. The authors established that the average bacterioplankton abundance/biomass in the upper 50 m layer varied between 0.4 × 10^6^ cells·mL^−1^/66 mg·m^−3^ (ArW) and 0.6 × 10^6^ cells·mL^−1^/41 mg·m^−3^ (AW), in mid-November 2013 [26]. Our study revealed lower values of the total bacterial biomass in the upper 50 m layer, while the bacterial abundance was comparable with previous autumn estimates [26]. In the northern Kara Sea (St. Anna Through region), the autumn abundance of marine bacteria in the surface layer was reported to be much lower [61,62], in comparison to our data, but the total bacterial biomass was comparable [61]. Spatial and seasonal differences may be explained by climatic variations, local hydrology, nutrient dynamics, and abundance of phytoplankton and zooplankton. In particular, previous studies conducted in the Barents Sea were performed during a warmer period (2010–2016) [24,25,26]. Other studies were focused on the spring and summer seasons, which are considered to be the most productive periods in the year, when biomass of phytoplankton was the highest [21,22,23]. The lower abundance of autumn bacterioplankton, in the northern Kara Sea, relative to our study, may be explained by oligotrophic conditions [62].

Virioplankton abundance in the Barents Sea, also, demonstrated seasonal fluctuations, with the highest values occurring in the summer period. In the central Barents Sea, the number of virus-like particles ranged between 0.66 × 10^6^ and 8.6 × 10^6^ particles·mL^−1^ [21]. Similar counts were reported for AW, while in ArW, virioplankton abundance tended to be lower [24,25]. Autumn abundances of viruses in the upper 50 m layer in the Barents Sea [26] were similar to those found in the summer period [24,25]. Our surface values were in good accordance, with previously detected summer and autumn estimations. In the northern Kara Sea, the mean abundance of virioplankton in autumn [63] was much lower compared to our data, which may, also, be explained by oligotrophic conditions in the region. In other Arctic regions, namely, the Laptev Sea and the East Siberian Sea, autumn virioplankton counts were similar to the mean values recorded in our study [64,65].

We detected a positive significant correlation between the abundance of viruses and bacterial stock, and this pattern appears to be common in the Arctic marine environment [20]. The variability of viral abundance is, largely, influenced by bacteria, pelagic microalgae, protists, and zooplankton [1,12]. In the Arctic, phytoplankton assemblages, during the spring-bloom succession, actively consume nutrients to grow and develop, and they produce organic matter, which is utilized by marine bacteria, promoting subsequent viral infection and viral abundance in seawater [20,66]. This pattern can be detected by analyzing VBR. The maximum VBR levels are typical, for regions with higher bacterial abundance and biomass. Previous investigations in the Barents Sea have documented a tendency of summer VBR to be greater, in regions with an enhanced number of marine bacteria [24,25]. During autumn and winter periods, VBR decreased, due to a drop in the total microbial stock in the Barents and Kara seas [26,63]. Our VBR estimations were higher, compared to summer values [24,25], suggesting a higher impact of viruses on bacterioplankton, after the main productive season. We revealed that contact rates (R) between viruses and bacteria significantly decreased with depth, while an inverse relationship was detected in the case of AVB. Our finding suggests that viral particles were associated, mainly, with smaller bacterial cells occurring in the upper layers, although it might be expected that larger bacteria would be preferable for viruses, due to a larger surface for attachment. However, small bacterial cells seem, likely, to be more accessible, owing to their greater abundance. Larger cells were concentrated in the bottom layers and had lower densities.

Results of the present study showed patchiness in the horizontal distribution of bacteria and viruses in the Barents Sea. This is a well-documented phenomenon, reflecting the discontinuous distribution of individual organisms in aquatic environments [59]. Aggregation of plankton may be connected with frontal zones, local eddies, environmental gradients, food stocks, predator abundance, and biotic interactions [59]. Hierarchical cluster analysis indicated a low degree of dissimilarity between sampling stations, and NMDS ordination based on microbial parameters showed a high stress value. Moreover, ANOSIM indicated non-significant differences in bacterioplankton and virioplankton, between BSW and ArW. Therefore, our hypothesis regarding the association of microbial plankton to water masses must be rejected. Nevertheless, we revealed a clear vertical pattern in the distribution of marine microbes, with minimum parameters being registered in the bottom layer. AVB, also, increased with depth, and larger bacteria were accumulated near the seafloor. SIMPER indicated the importance of marine viruses, to distinguish depth layer by microbial parameters. Greater abundances of marine bacteria and viruses, in the upper and intermediate layers, are a common feature reported for various Arctic regions [60,61,62,63,64,65], including the Barents Sea [21,22,23,24,25,26]. This pattern can be explained by the nutrient distribution and local hydrological properties.

### 4.3. Environmental Control of Microbial Plankton

The RDA revealed that a set of environmental variables (temperature, salinity, nutrients, chlorophyll *a*, and zooplankton stock) accounted for 61% of the total fluctuation in microbial plankton. Three parameters were found to be significantly associated with bacterioplankton and virioplankton. The most important factor affecting the distribution of microbial plankton was nitrate concentration. Negative correlations between nutrient supply and microbial abundance are considered to be, indirectly, connected with phytoplankton. Chlorophyll *a* was found to be one of the main drivers, controlling both abundance and biomass of marine microbes in the Arctic, during the spring and summer seasons [21,22,62,64,65]. In our study, chlorophyll *a* values were low, suggesting that phytoplankton biomass was lower than in the most productive season. Nevertheless, microalgae seem to be important organisms, utilizing inorganic nutrients in the upper layer. On the other hand, there were great concentrations of nitrate and phosphate in the bottom layer, where bacterial abundance was significantly lower. This, also, might explain negative correlations between inorganic nutrients and microbial plankton. The enhanced supply of organic matter increases bacterial growth and leads to an increase in viral abundance in the marine environment [66,67]. Phytoplankton are the main source of organic carbon for microbial plankton, in the spring and summer periods, while other organisms may be important after the main productive period [11]. Microzooplankton and copepods can, rapidly, recycle and regenerate both dissolved organic and inorganic nutrients, through feeding, excretion, and releasing of fecal pellets in autumn [68]. Moreover, zooplankton, rather than phytoplankton, appear to be the key autochthonous source of bacterial substrates.

In contrast, virioplankton abundance and VBR negatively correlated with zooplankton biomass. This result is in contrast to other studies reporting an increase in viral abundance with carbon biomass, in the spring and summer periods [60,64]. It is more likely that these differences might be related to seasonal fluctuations in the distribution of plankton animals and their vertical migrations as well as the dominance of small-sized bacterioplankton in our study. Viruses are associated with suspended and particulate particles as well as small heterotrophic plankton [36]. Larger zooplankters, often, consume microzooplankton in the autumn and winter seasons [59,68], thus reducing the number of potential hosts for viruses. The observed pattern may, also, be explained by a direct influence of the smaller zooplankton, on potential hosts for marine viruses. Nauplii and omnivorous copepods might ingest detritus and particulate organic matter [68], a potential surface for marine viruses, and this might result in reduced virioplankton abundance. An alternative explanation is food selectivity of the larger zooplankton, which can consume different-sized fractions of microzooplankton [68]. They might prefer to ingest larger cells of protists, while the remaining nanoplankton and picoplankton seemed to be responsible for grazing marine bacteria and virus-like particles. Additionally, some modeling studies [69,70] have shown that zooplankton organisms are able to avoid virally infected phytoplankton and this, therefore, might be reflected as a negative correlation between the total zooplankton abundance/biomass and viral density. Our study and previous investigations dealing with autumn and winter Arctic zooplankton [29,30] highlighted the dominance of small copepods in the total zooplankton abundance, most of which are found to be omnivorous, preferring to ingest protozooplankton in the periods of low phytoplankton availability [68].

Salinity, together with water temperature, explained part of the variation in microbial abundance in the Barents Sea. Bacterioplankton and virioplankton abundances demonstrated a tendency to be lower with salinity, and an opposite pattern was revealed, in the case of water temperature. This result can be interpreted, considering the vertical distribution of microbes. Greater densities were located in warmer surface waters, while bottom high-saline and colder waters showed lower microbial counts. The activity of marine bacteria is known to be temperature-dependent [71] and, therefore, their growth would be slower at lower temperatures, near the bottom. Other investigations documented that temperature and salinity play a minor role in determining spatial variations, in the microbial abundance and biomass in the Arctic regions [9,22,72]. Thus, it is the concentration of organic and inorganic nutrients that are the most responsible factors affecting microbial plankton distribution in the Barents Sea, but sources of organic carbon, obviously, differ seasonally.

## 5. Conclusions

Microbial communities are, strongly, involved in biogeochemical cycles and represent a major link in the marine food webs and carbon fluxes. Plankton assemblages are considered good indicators of environmental impacts. The Arctic Ocean is the region, where clear climatic fluctuations have been reported. Studying plankton in the Arctic provides valuable data, to predict overall biotic responses to climatic changes. The present study was focused on a less-investigated region of the Arctic Ocean—the northeastern Barents Sea. Bacterioplankton abundance and biomass as well as concentration of virus-like particles demonstrated mosaic horizontal distribution in the autumn period. There were no clear associations of the microbial variables, with two main water masses (colder Arctic Water and warmer Barents Sea Water). A clear vertical pattern in microbial abundance was evident, with the highest concentrations in the upper layers. Small-sized cells were the most dominant group of the bacterioplankton, and average bacterial-cell volume tended to increase, near the seafloor. Bacterial abundance was lower than in other seasons and regions of the Barents Sea, while viral abundance was comparable. Bacterioplankton abundance was positively correlated with concentrations of viruses, indicating close relationships between these groups of microbes. Nutrients and organic carbon of zooplankton, together with hydrological variables, explained most of the spatial variability in microbial parameters.

Our study expands current knowledge on the structure of pelagic ecosystems in the Arctic and provides baseline information on marine microbes in the autumn season. This investigation may be used in future research, focusing on the ecology of Arctic plankton assemblages, during the period of global environmental changes.

## Figures and Tables

**Table 1:** Location and hydrology of sampling stations in the northeastern Barents Sea, in October 2020 [27].

ID	Date	N	E	Depth, m	Temp	Sal	Water Mass
7	08.10.2020	77°23′	67°44′	360	−1.28–2.51	33.95–34.89	BSW
8	09.10.2020	77°50′	66°46′	330	−0.98–1.55	34.21–34.94	BSW
9	09.10.2020	78°19′	65°41′	365	−1.50–1.43	34.08–34.88	ArW
10	09.10.2020	78°47′	64°32′	355	−1.27–0.98	33.92–34.84	ArW
11	09.10.2020	79°15′	63°16′	255	−1.54–0.61	33.49–34.83	ArW
12	10.10.2020	79°25′	59°24′	247	−1.79–1.07	33.72–34.83	ArW
13	10.10.2020	78°56′	60°41′	192	−1.66–0.09	33.57–34.81	ArW
14	10.10.2020	78°26′	61°55′	290	−1.33–1.41	33.88–34.84	ArW
15	10.10.2020	77°57′	63°27′	340	−1.10–2.05	34.02–34.85	ArW

Note. Temp—temperature (°C), Sal—salinity. Water mass: BSW—Barents Sea Water, ArW—Arctic Water.

**Table 2:** Environmental parameters and microbial variables in different water masses and sampling layers in the northeastern Barents Sea, in autumn 2020.

Parameter	A Surface (0–25 m)		B Intermediate (50–100 m)		C Bottom (192–365 m)	
	Range	Mean ± SE	Range	Mean ± SE	Range	Mean ± SE
	Arctic Water					
NB, 10^5^ cell·mL^−1^	3.33–9.25	5.67 ± 0.35 ^C^	2.96–10.68	6.12 ± 0.77 ^C^	2.27–4.09	3.20 ± 0.27 ^AB^
BB, mgC·m^−3^	3.4–12.5	6.2 ± 0.6 ^C^	4.1–12	7.7 ± 0.8 ^C^	2.9–8.2	4.5 ± 0.7 ^AB^
VBR	1–28	13 ± 2	7–14	11 ± 1	4–14	9 ± 1
ABV, µm^3^	0.02–0.074	0.037 ± 0.003 ^BC^	0.033–0.085	0.049 ± 0.005 ^A^	0.031–0.136	0.055 ± 0.014 ^A^
R	0.8–7.1	4.0 ± 0.5	3.0–9.6	5.7 ± 0.7	0.3–1.4	0.9 ± 0.2 ^AB^
NV, 10^6^ particles·mL^−1^	0.9–15	7.1 ± 1.0 ^C^	5.7–10.1	7.6 ± 0.5 ^C^	1.4–4.3	2.9 ± 0.5 ^AB^
Temperature, °C	−0.2–2.0	1.0 ± 0.2 ^BC^	−1.7–0.4	−1.2 ± 0.2 ^A^	−1.3–−0.8	−1.0 ± 0.1 ^A^
Salinity	33.49–34.08	33.84 ± 0.06 ^BC^	34.06–34.74	34.63 ± 0.06	34.73–34.84	34.81 ± 0.02
PO_4_, µM	0.0–0.2	0.1 ± 0.0 ^BC^	0.0–0.7	0.4 ± 0.1 ^AC^	0.3–0.9	0.6 ± 0.1 ^AB^
O_2_, mL·L^−1^	7.8–8.0	8.0 ± 0.0 ^C^	7.0–8.2	7.6 ± 0.1 ^C^	6.8–7.0	6.9 ± 0.0 ^AB^
NO_3_, µM	0.1–1.3	0.6 ± 0.1 ^BC^	0.4–16.1	8.4 ± 1.4 ^AC^	15–18.1	17.2 ± 0.4 ^AB^
Chl *a*, mg·m^−3^	0.19–0.36	0.28 ± 0.02	–	–	–	–
ZA, ind.·m^−3^ (0–100 m)	752–1278, 1067 ± 35					
ZB, mgC·m^−3^ (0–100 m)	0.8–11.8, 6.3 ± 0.6					
	Barents Sea Water					
NB, 10^5^ cell·mL^−1^	4.46–6.36	5.47 ± 0.27 ^C^	4.33–5.80	5.06 ± 0.73 ^C^	2.20–3.53	2.87 ± 0.67 ^AB^
BB, mgC·m^−3^	4.6–9.6	6.1 ± 0.8 ^C^	5.6–6.1	5.8 ± 0.2 ^C^	2.7–5.5	4.1 ± 1.4 ^AB^
VBR	0–15	11 ± 2	14–15	15 ± 1 ^C^	10–11	10 ± 0
ABV, µm^3^	0.026–0.056	0.037 ± 0.005 ^C^	0.03–0.051	0.041 ± 0.01 ^C^	0.042–0.059	0.051 ± 0.008 ^AB^
R	2.2–6.1	4.3 ± 0.7 ^C^	2.8–4.7	3.7 ± 0.9 ^C^	0.5–1.4	0.9 ± 0.4 ^AB^
NV, 10^6^ particles·mL^−1^	4.4–9.1	7.0 ± 0.7 ^C^	6.0–8.9	7.5 ± 1.4 ^C^	2.4–3.6	3.0 ± 0.6 ^AB^
Temperature, °C	1.5–2.5	2.0 ± 0.2 ^BC^	−0.4–−0.2	–0.3 ± 0.1 ^AC^	−1–−0.9	–0.9 ± 0.0 ^AB^
Salinity	33.95–34.27	34.14 ± 0.06 ^BC^	34.62–34.7	34.66 ± 0.04 ^A^	34.83–34.83	34.83 ± 0.01 ^A^
P-PO_4_, µM	0.1–0.1	0.1 ± 0.0 ^BC^	0.3–0.5	0.4 ± 0.1 ^AC^	0.6–0.7	0.7 ± 0.0 ^AB^
O_2_, mL·L^−1^	7.6–7.8	7.7 ± 0.0 ^C^	7.0–7.7	7.4 ± 0.3 ^C^	6.8–6.9	6.8 ± 0.0 ^AB^
N-NO_3_, µM	0.7–1.4	0.9 ± 0.1 ^BC^	2.5–3.9	3.2 ± 0.7 ^AC^	18.1–18.7	18.4 ± 0.3 ^BC^
Chl *a*, mg·m^−3^	0.18–0.32	0.27 ± 0.03	–	–	–	–
ZA, ind.·m^−3^ (0–100 m)	930–1008, 970 ± 13					
ZB, mgC·m^−3^ (0–100 m)	1.5–1.9, 1.7 ± 0.1					

Note. NB—bacterial abundance, BB—bacterial biomass, VBR—the ratio of viral to bacterial abundance, ABV—average bacterial cell volume, R—contact rate between viruses and bacteria, NV—viral abundance. Chl *a*—surface chlorophyll *a* concentration (mg·m^−3^), PO_4_—phosphate concentration (µM), O_2_—dissolved oxygen concentration (mL·L^−1^), NO_3_—nitrate concentration (µM), ZA—zooplankton abundance in the upper 100 m layer (ind.·m^−3^), ZB—zooplankton biomass in the upper 100 m layer (mgC·m^−3^). The letters show significant differences between sampling layers (*p* < 0.05).

**Table 3:** Results of Monte Carlo permutation test in RDA: list of environmental variables affected microbial plankton in the northeastern Barents Sea, in autumn 2020.

Variable	LambdaA	P	F	Variance Inflation Factor (VIF)	Explained Variation, %
Nitrate	0.27	0.001	10.23	9.455	27
Zooplankton biomass	0.12	0.005	5.44	4.959	12
Salinity	0.06	0.045	3.01	5.501	6
Temperature	0.05	0.069	2.77	5.368	5
Dissolved oxygen	0.05	0.068	2.65	9.845	5
Phosphate	0.05	0.11	2.39	8.011	5
Zooplankton abundance	0.01	0.514	0.7	2.702	1
Chlorophyll *a*	0.001	0.913	0.14	1.585	<1

**Table 4:** Pearson’s correlation coefficients between environmental parameters and microbial variables in the northeastern Barents Sea, in autumn 2020.

Parameter	Temperature	Salinity	PO_4_	O_2_	NO_3_	Chl *a*	ZA	ZB
NB, 10^5^ cell·mL^−1^	0.26	**−0.50**	**−0.44**	**0.64**	**−0.63**	−0.03	0.08	0.19
BB, mgC·m^−3^	−0.02	−0.28	−0.23	0.34	−0.34	0.15	0.25	0.28
NV, 10^6^ particles·mL^−1^	**0.40**	−0.17	−0.29	**0.46**	**−0.52**	−0.26	−0.21	**−0.37**
ABV, µm^3^	0.29	0.13	−0.04	0.10	−0.18	−0.26	−0.29	**−0.54**
VBR	−0.35	0.27	0.24	−0.35	**0.38**	0.31	0.32	0.15
R	**0.42**	−0.35	**−0.43**	**0.60**	**−0.65**	−0.13	−0.07	−0.14

Note. NB—bacterial abundance, BB—bacterial biomass, VBR—the ratio of viral to bacterial abundance, ABV—average bacterial cell volume, R—contact rate between viruses and bacteria, NV—viral abundance. T—temperature (°C), Sal –salinity, Chl *a*—surface chlorophyll *a* concentration (mg·m^−3^), PO_4_—phosphate concentration (µM), O_2_—dissolved oxygen concentration (mL·L^−1^), NO_3_—nitrate concentration (µM), ZA—zooplankton abundance in the upper 100 m layer (ind.·m^−3^), ZB—zooplankton biomass in the upper 100 m layer (mgC·m^−3^). Bold font indicates significant *p*-values, confirmed by the Benjamini–Hochberg procedure.

## Data Availability

The data are available on request, from the corresponding author.

## Supplementary Materials

The following are available online at https://www.mdpi.com/article/10.3390/biology11060845/s1, Figure S1: T-S diagram of different water masses in the northeastern Barents Sea, in autumn 2020. BSW—Barents Sea Water, ArW—Arctic Water. Figure S2: Relationship between bacterial abundance (NB) and phosphate concentration in the northeastern Barents Sea, in autumn 2020. R^2^—determination coefficient. N = 30. Figure S3: Relationship between bacterial abundance (NB) and nitrate concentration in the northeastern Barents Sea, in autumn 2020. R^2^—determination coefficient. N = 30. Figure S4: Relationship between viral abundance (NV) and water temperature in the northeastern Barents Sea, in autumn 2020. R^2^—determination coefficient. N = 30. Figure S5: Relationship between viral abundance (NV) and nitrate concentration in the northeastern Barents Sea, in autumn 2020. R^2^—determination coefficient. N = 30. Figure S6: Relationship between viral abundance (NV) and zooplankton biomass in the northeastern Barents Sea, in autumn 2020. R^2^—determination coefficient. N = 30. Figure S7: Relationship between VBR (the ratio of viral to bacterial abundance) and zooplankton biomass in the northeastern Barents Sea, in autumn 2020. R^2^—determination coefficient. N = 30. Figure S8: Relationship between ABV (average bacterial cell volume) and nitrate concentration in the northeastern Barents Sea, in autumn 2020. R^2^—determination coefficient. N = 30. Figure S9: Relationship between R (contact rate between viruses and bacteria) and water temperature in the northeastern Barents Sea, in autumn 2020. R^2^—determination coefficient. N = 30. Figure S10: Relationship between R (contact rate between viruses and bacteria) and phosphate concentration in the northeastern Barents Sea, in autumn 2020. R^2^—determination coefficient. N = 30. Figure S11: Relationship between R (contact rate between viruses and bacteria) and nitrate concentration in the northeastern Barents Sea, in autumn 2020. R^2^—determination coefficient. N = 30.
