# Supplementary material for: Pelagic Bacteria and Viruses in a High Arctic Region: Environmental Control in the Autumn Period
Source: Biology (Basel). 2022 May 31;11(6):845. doi: 10.3390/biology11060845 (PMC9220044; doi:10.3390/biology11060845)
Supplement: Supplementary file 1 [file biology-11-00845-s001.zip › biology-1715159-supplementary.pdf]

Supplementary material. Figures S1–S11.
Pelagic bacteria and viruses in a high Arctic region: Environmental control in the autumn period
Vladimir G. Dvoretsky, Marina P. Venger, Anastasya V. Vashchenko, Tatyana M. Maksimovskaya, Tatyana G. Ishkulova and Veronika V. Vodopianova
Biology 2022.

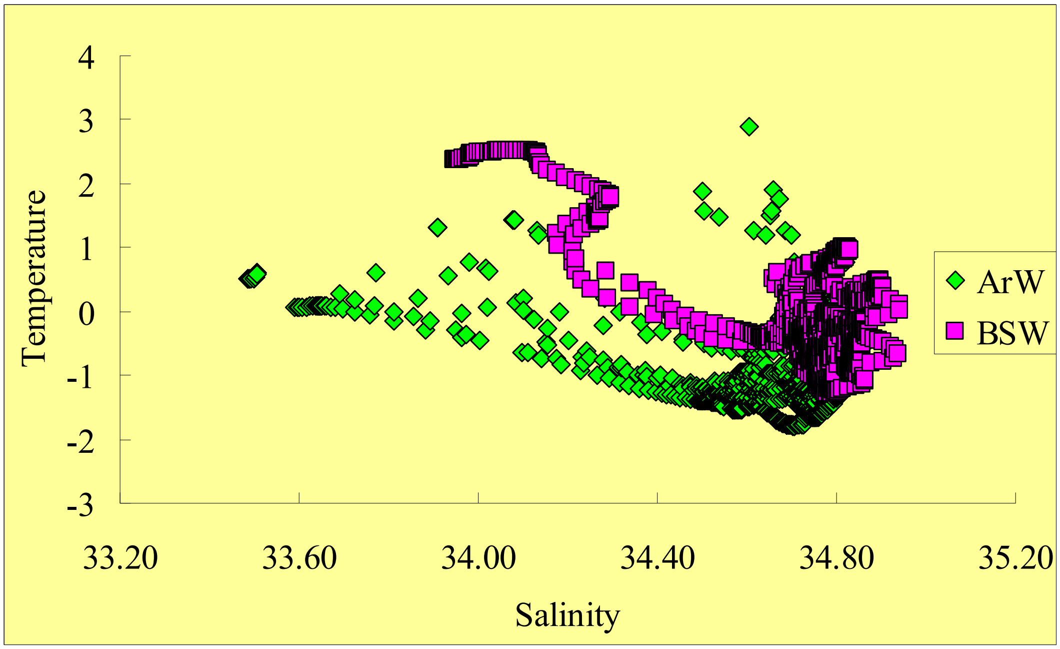

Figure S1. T-S diagram of different water masses in the north-eastern Barents Sea in autumn 2020. BSW – Barents Sea Water, ArW – Arctic Water.

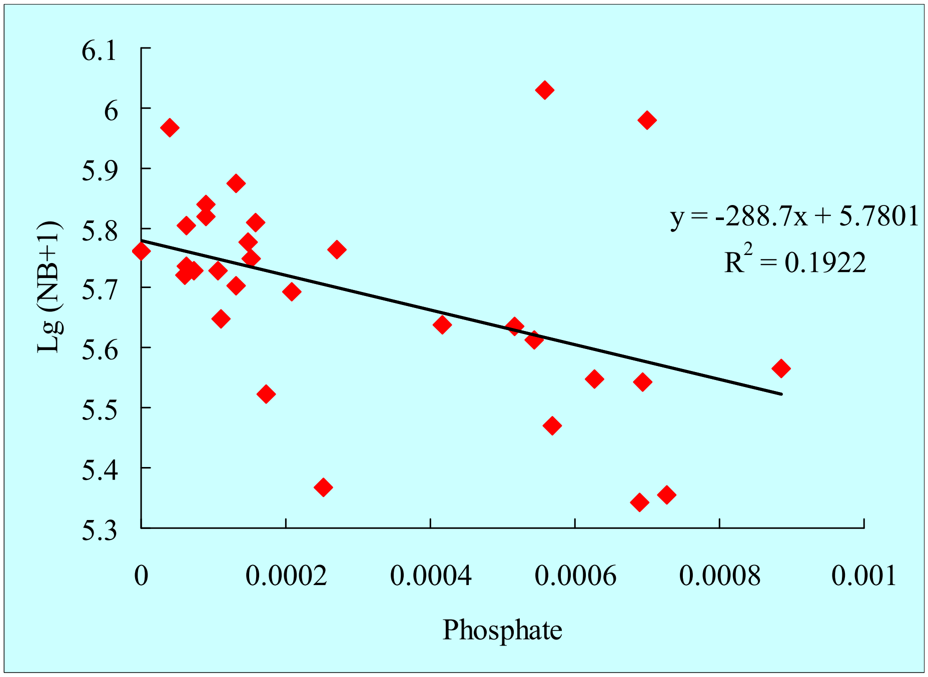

Figure S2. Relationship between bacterial abundance (NB) and phosphate concentration in the north-eastern Barents Sea in autumn 2020. R^2 – determination coefficient. N = 30.

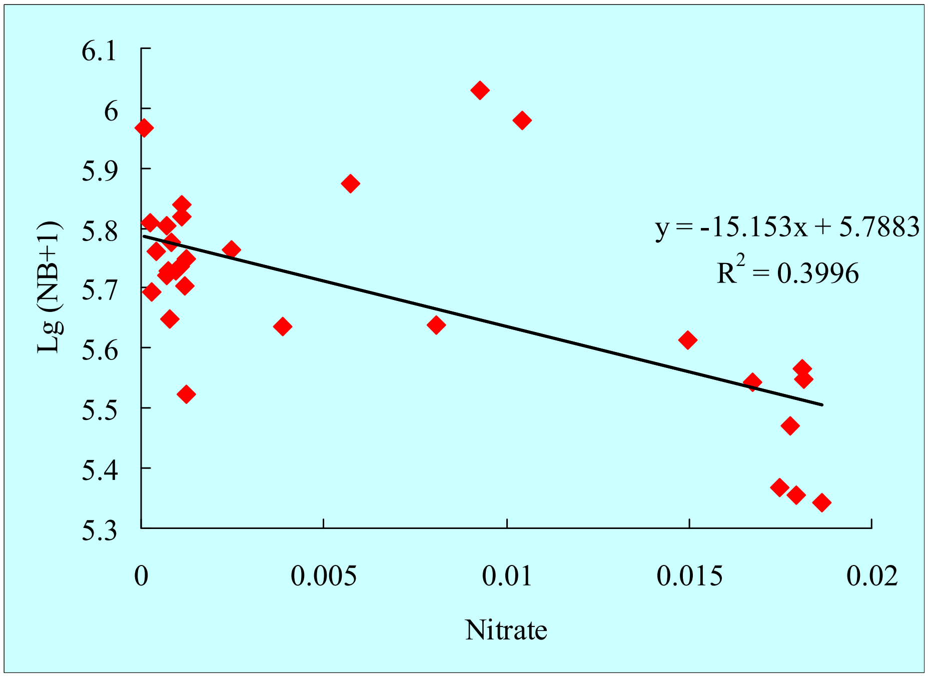

Figure S3. Relationship between bacterial abundance (NB) and nitrate concentration in the north-eastern Barents Sea in autumn 2020. R^2 – determination coefficient. N = 30.

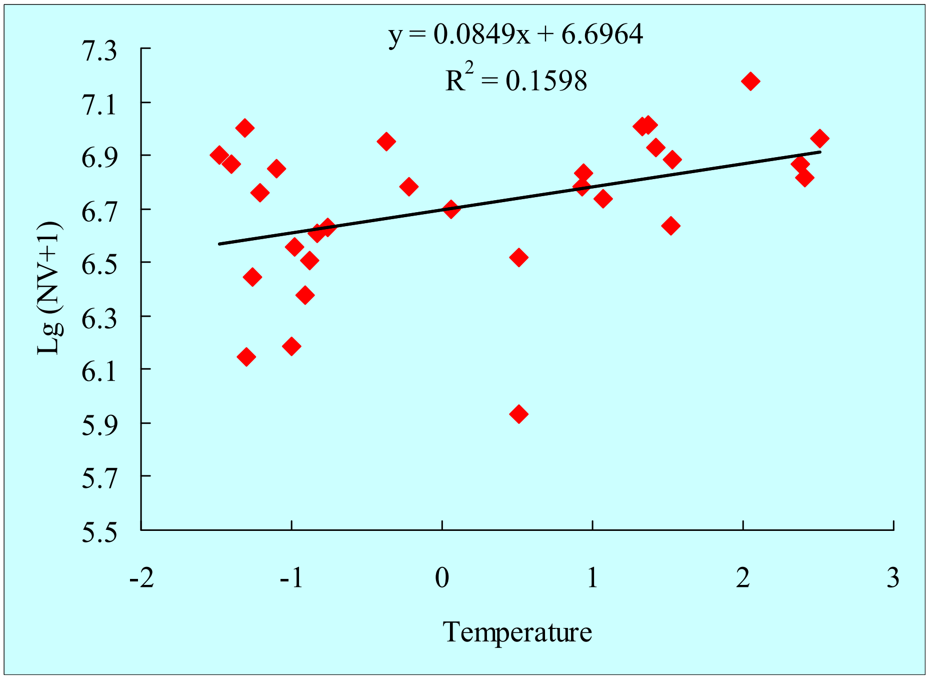

Figure S4. Relationship between viral abundance (NV) and water temperature in the north-eastern Barents Sea in autumn 2020. R^2 – determination coefficient. N = 30.

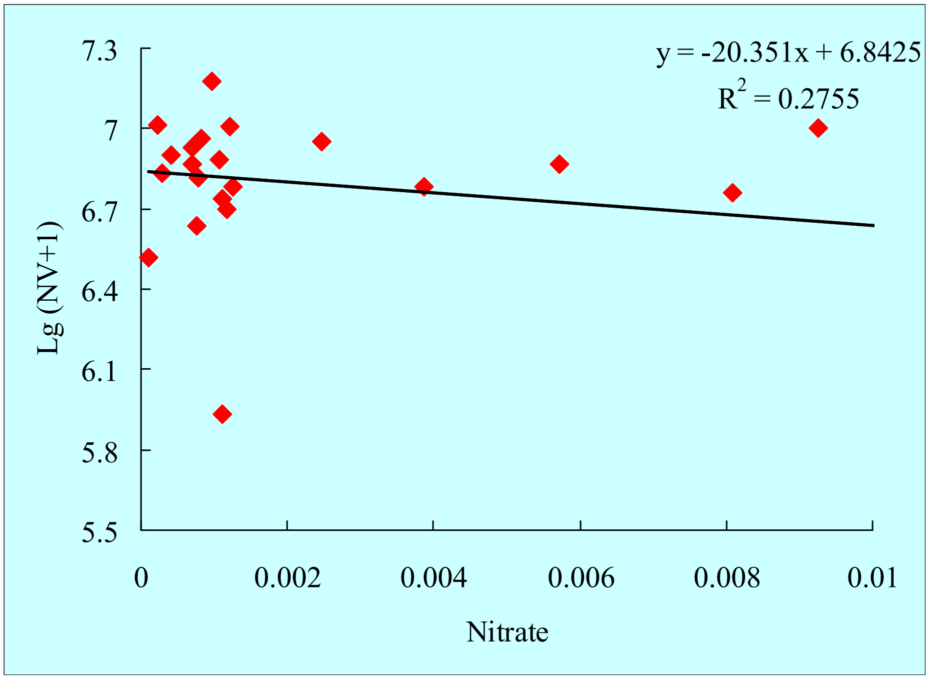

Figure S5. Relationship between viral abundance (NV) and nitrate concentration in the north-eastern Barents Sea in autumn 2020. R^2 – determination coefficient. N = 30.

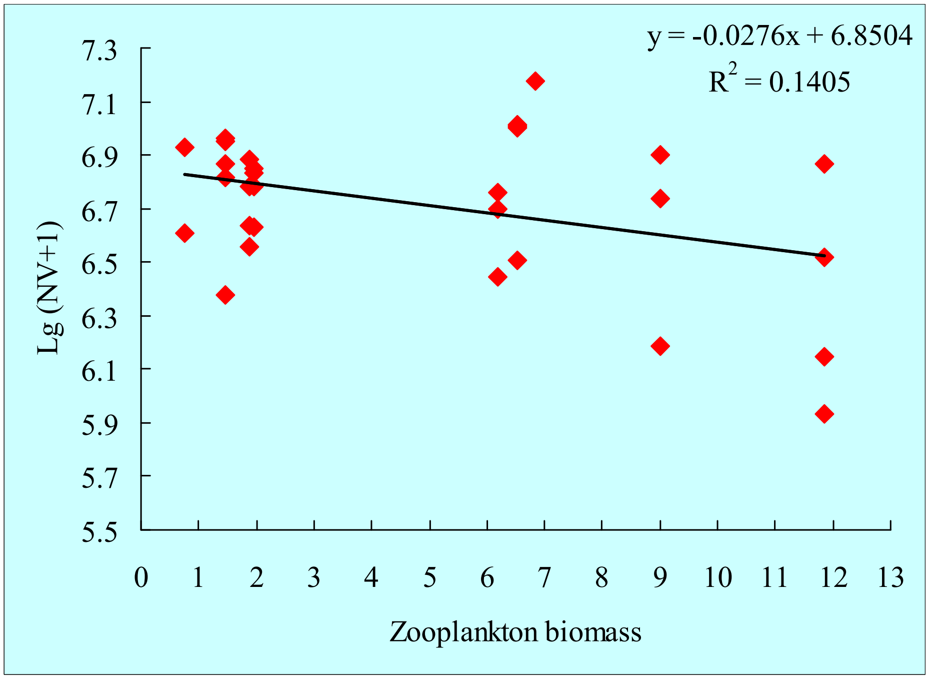

Figure S6. Relationship between viral abundance (NV) and zooplankton biomass in the north-eastern Barents Sea in autumn 2020. R^2 – determination coefficient. N = 30.

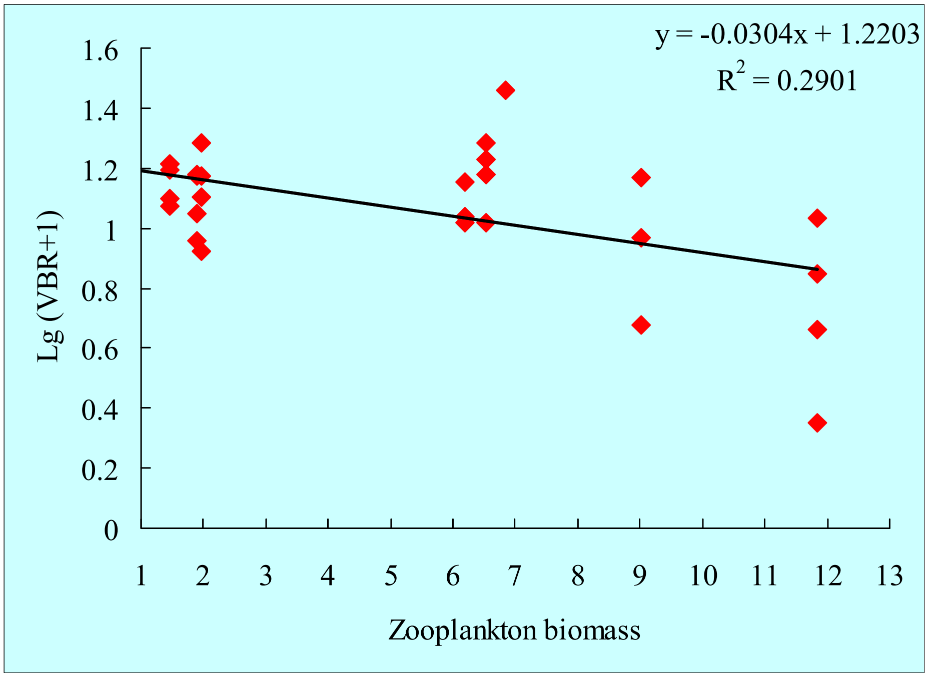

Figure S7. Relationship between VBR (the ratio of viral to bacterial abundance) and zooplankton biomass in the north-eastern Barents Sea in autumn 2020. R^2 – determination coefficient. N = 30.

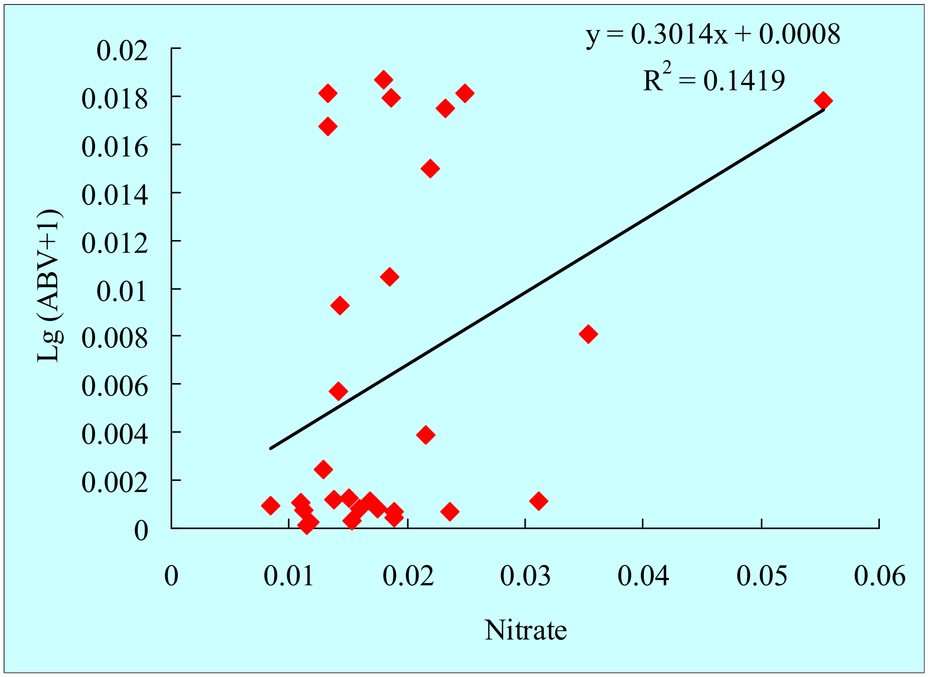

Figure S8. Relationship between ABV (average bacterial cell volume) and nitrate concentration in the north-eastern Barents Sea in autumn 2020. R^2 – determination coefficient. N = 30.

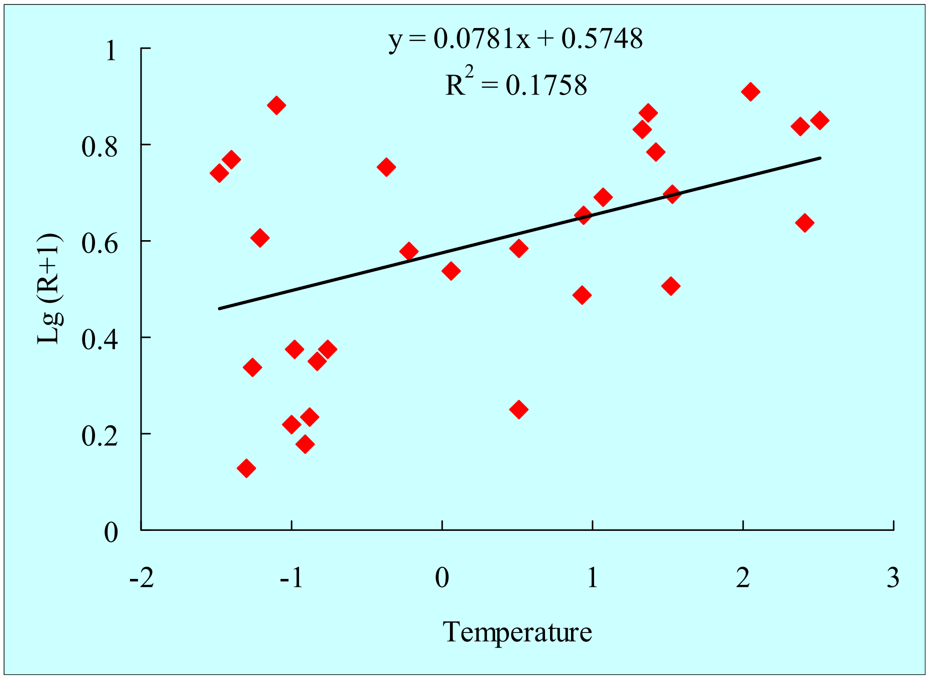

Figure S9. Relationship between R (contact rate between viruses and bacteria) and water temperature in the north-eastern Barents Sea in autumn 2020. R^2 – determination coefficient. N = 30.

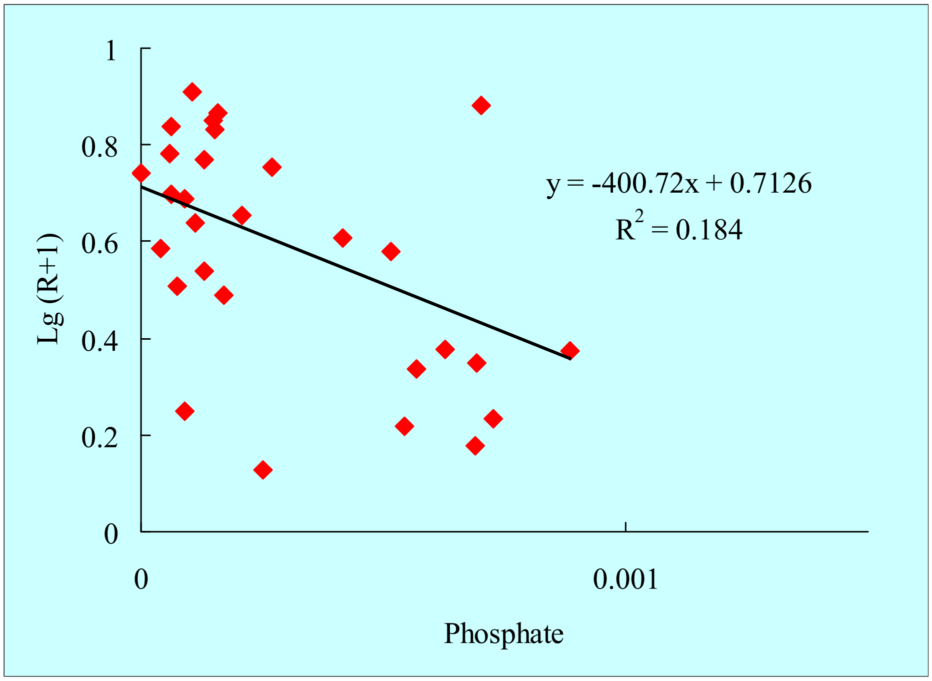

Figure S10. Relationship between R (contact rate between viruses and bacteria) and phosphate concentration in the north-eastern Barents Sea in autumn 2020. R^2 – determination coefficient. N = 30.

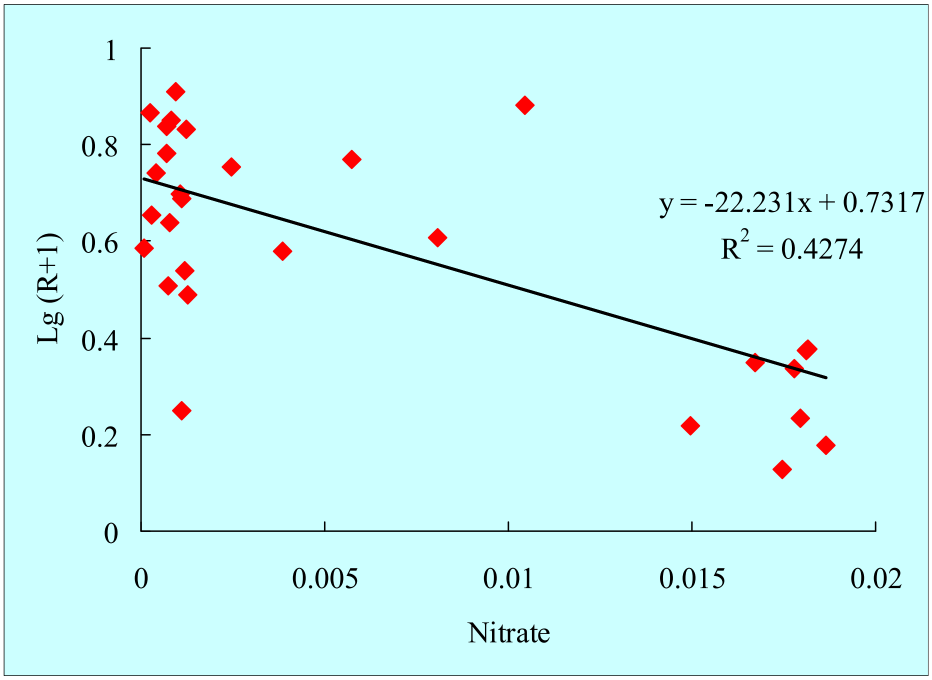

Figure S11 . Relationship between R (contact rate between viruses and bacteria) and nitrate concentration in the north-eastern Barents Sea in autumn 2020. R^2 – determination coefficient. N = 30.
